# Nutrition and physical activity intervention for families with familial hypercholesterolaemia: protocol for a pilot randomised controlled feasibility study

**DOI:** 10.1186/s40814-020-00584-3

**Published:** 2020-04-02

**Authors:** Fiona J. Kinnear, Julian P. Hamilton-Shield, David J. Stensel, Graham Bayly, Aidan Searle, Alice E. Thackray, Fiona E. Lithander

**Affiliations:** 1 NIHR Bristol Biomedical Research Centre (Nutrition Theme), University Hospitals Bristol NHS Foundation Trust and the University of Bristol, Bristol, UK; 2 National Centre for Sport and Exercise Medicine, School of Sport, Exercise and Health Sciences, Loughborough University, Epinal Way, Loughborough, UK; 3 University Hospitals of Leicester NHS Trust, Infirmary Square, Leicester, UK; 4 Department of Clinical Biochemistry, University Hospitals Bristol NHS Foundation Trust, Bristol, UK

**Keywords:** Familial hypercholesterolaemia, Diet, Physical activity, Cardiovascular disease, Intervention, Dietetics, Paediatrics, Primary prevention, Qualitative, Feasibility

## Abstract

**Background:**

Untreated heterozygous familial hypercholesterolaemia (FH) causes high low-density lipoprotein cholesterol (LDL-C) levels and increased cardiovascular disease (CVD) risk. Despite pharmacological treatment, many treated individuals remain at higher CVD risk than non-affected individuals. This may be due to LDL-C targets not being met and presence of other CVD risk factors. Adhering to dietary and physical activity (PA) recommendations developed for individuals with FH may further reduce CVD risk. However, there is insufficient research to support the efficacy of adhering to these guidelines on LDL-C and other CVD risk factors. The need for studies to investigate the effectiveness of nutrition and PA interventions in the FH population has been widely recognised and recommended. This paper describes the protocol of a pilot, randomised controlled trial designed to evaluate the feasibility and acceptability of a specifically developed nutrition and PA intervention aimed at improving the dietary intakes and PA levels of families with FH.

**Methods:**

A two-arm randomised waitlist-controlled pilot trial will be conducted across three National Health Service (NHS) sites in England, UK. Twenty-four young people with FH, aged 10–18 years, and their affected parent, will be recruited and randomly assigned to the intervention or waitlist and usual care control. The primary aim is to provide evidence for the feasibility and acceptability of delivering the intervention, explored quantitatively (rates of recruitment, retention and outcome measure completeness) and qualitatively (qualitative interviews). The secondary aim is to provide evidence for the potential efficacy of the intervention on dietary intake, PA, sedentary time, body composition, CVD risk factors and quality of life determined at baseline and endpoint assessments. The intervention will involve an hour-long consultation with a dietitian at baseline and four follow-up contacts across the 12-week intervention. It has been specifically developed for use with individuals with FH and incorporates behavioural change techniques to target identified enablers and barriers to adherence in this population.

**Discussion:**

This trial will estimate the feasibility and acceptability of the nutrition and PA intervention delivered to young people and parents with FH. If appropriate, this study can be used to inform the design of an adequately powered definitive trial.

**Trial registration:**

ISRCTN, ISRCTN24880714. Registered 07/06/2018, http://www.isrctn.com/ISRCTN24880714.

## Background

Heterozygous familial hypercholesterolaemia (FH) is an autosomal dominant hereditary disorder, characterised by markedly elevated levels of low-density lipoprotein cholesterol (LDL-C) from birth [1]. The lifelong exposure to elevated LDL-C levels confers a substantially increased risk of premature cardiovascular disease (CVD) and associated mortality [2] to the 1 in 250 people affected worldwide [3]. While the introduction of pharmacological treatment has dramatically reduced the incidence of CVD in individuals with FH, they remain at increased risk [4–7]. The residual CVD risk may be attributed to the large number of adults and young people that do not reach LDL-C treatment targets, even on maximally tolerated doses [8, 9]. Additionally, the presence of other risk factors such as hypertension, obesity and type II diabetes have been shown to be independently associated with their CVD risk [6, 10–14]. Efforts are currently focussed on improving the low overall detection of this disorder, which is estimated to be identifying only 1–15% of those at risk worldwide [15]. It is therefore crucial to optimise the treatment provided to this rapidly increasing vulnerable patient population group.

Lifestyle advice is considered an important adjuvant to pharmacological treatment for individuals with FH. All individuals are recommended to receive individualised advice about diet, physical activity and the maintenance of a healthy weight from a healthcare professional (HCP) with specific expertise [16–18]. Dietary and physical activity recommendations are based on the same principles as advised for the general population, with additional emphasis to reduce intakes of foods high in fat, saturated fat and cholesterol and to consume foods with LDL-C-lowering effects such as plant phytosterols/phytostanol s[16–18].. Despite these recommendations, comprehensive systematic reviews have concluded that there is insufficient data to make any conclusions about the effectiveness of any dietary or physical activity intervention upon CVD risk or surrogate outcomes in adults or young people with FH [19, 20]. Extrapolating the results of such interventions carried out in other patient populations is inappropriate as this does not allow for consideration of the unique physiological and psychological factors present in individuals with FH. Randomised controlled trials (RCTs) to investigate the effectiveness of dietary and physical activity interventions within FH cohorts have been recommended to address this research gap [18–21].

An understanding of how and why individuals with FH display certain behaviours and an awareness of the factors influencing their decisions to adhere to treatment should be considered in the development of an intervention. Adherence to lifestyle recommendations in affected individuals is sub-optimal whereby less than 50% of adults reported following the recommended lifestyle advice [22]. Additionally, overweight and obesity has been reported in cohorts of adults and young people with FH, suggesting that the advice is not being followed by all [9, 14, 23]. Given the chronic nature of FH, it is essential that an intervention enables individuals to make lifelong behavioural changes, rather than for the short duration of a clinical trial. As the greatest reduction in CVD risk is achieved when treatment is started at a young age [4, 24], young people stand to gain the most for receiving a lifestyle intervention. Given the increasing diagnostic rates and high prevalence of FH, any intervention to be evaluated should also be pragmatic and feasible to provide within the constraints of current healthcare services.

In the current research, we propose to develop and evaluate a family-based nutritional and physical activity intervention to enable young people with FH, and their affected parent, to achieve dietary and physical activity recommendations thus reducing their CVD risk. As there is currently insufficient evidence to support a large, full-scale RCT to evaluate such an intervention, a pilot RCT is needed as advised in the British Medical Research Council (MRC) framework for the development and evaluation of complex interventions [25, 26].

### Study aims and objectives

The primary aim of this study is to provide evidence for the feasibility and acceptability of conducting a future adequately powered, randomised controlled trial to evaluate a specifically designed nutrition and physical activity intervention in young people and their affected parent with FH. The primary objectives of the study are to explore the following questions:

1. Will young people and their parent be willing to participate in the proposed family-based intervention?
2. Will families accept randomisation to the control or intervention group, adhere to research methods in their allocated group and complete the study?
3. Are the research methods that are used to capture data fit for purpose?
4. Is there sufficient protocol fidelity in the intervention group?
5. Upon completion of the study, was the overall experience of the intervention and/or the study research processes positive and potentially reproducible in an adequately powered trial?

The secondary aim of this study is to estimate the potential efficacy of the intervention on the dietary intake, physical activity and sedentary time, body composition, selected CVD risk factors and quality of life of young people and their affected parent with FH.

## Methods

This protocol is reported in accordance with SPIRIT (Standard Protocol Items: Recommendations for Interventional Trials) guidance and a completed checklist is available in Additional File 1.

### Study design and setting

**Fig. 1:**
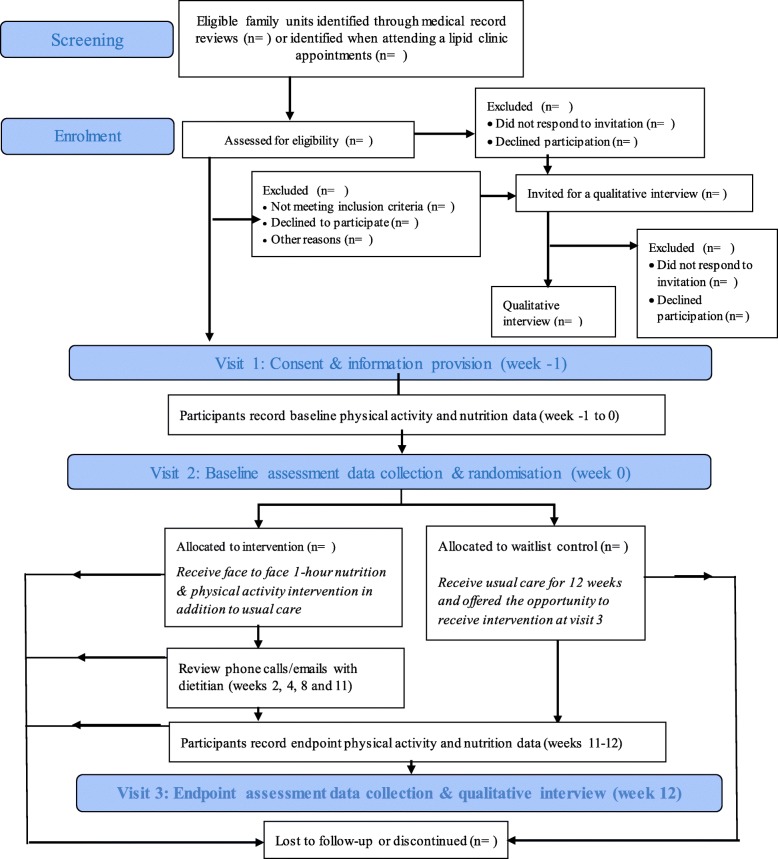
Study flow diagram

This pilot study is a two-arm, randomised waitlist-controlled trial comparing a specifically developed nutrition and physical activity intervention against usual-care waitlist control amongst young people with FH. This study aims to recruit family units, each comprised of a young person-parent dyad. This is in recognition of the autosomal dominant presentation of FH which means all affected young people will have one affected parent. This will also facilitate the proposed family-based delivery of the intervention. This study will be conducted across three National Health Service (NHS) Foundation Trust sites in England, UK: University Hospitals Bristol, Royal United Hospitals Bath and St. George’s University Hospitals London (Fig. 1).

### Study population

In this family-based intervention, young person(s) aged 10–18 years and their affected parent with FH will be invited to participate. A family unit will be comprised of at least one adult and one young person with FH, but there may also be family units in which additional young family members with FH take part.

#### Eligibility criteria

Individuals aged 10–18 years with a genetically confirmed clinical diagnosis of heterozygous FH who receive their care from the paediatric or adult lipid clinics at the three NHS Foundation Trust sites will be invited to join the study. Their parent (≥ 18 years), with a genetically confirmed clinical diagnosis of heterozygous FH, will also be invited to join the study.

Pregnant female patients, or those planning pregnancy, and individuals who are unable to give informed consent or assent for those aged 10–15 years are not eligible for participation. Patients with a diagnosis of homozygous FH and patients not established on their current treatment regimen for at least 1 month prior to recruitment to the study are not eligible for participation.

If a young person with FH wishes to join the study but their affected parent is deceased or does not want, or is not eligible to participate, then the young person will still be able to participate. In this instance, a non-affected parent or main carer of the young person will be invited to take part to facilitate the family-based aspect of the study if they wish. However, blood samples will not be collected from these non-affected participants. In instances where the non-affected parent or main carer also does not wish to take part, the young person with FH will still be able to participate on their own.

### Recruitment

#### Identification

All young people receiving care from paediatric or adult lipid clinic services at participating sites will be screened for eligibility. The eligibility of their affected parent will then be considered. This will be carried out by a member of the hospital care team and will be repeated every 3 months during the recruitment phrase. This will be conducted at both paediatric and adult clinics because young adults often transition to adult services before the age of 18 years.

#### Invitation to participate

A study invitation letter and an age-appropriate participant information sheet (PIS) will be sent to the families of young people who are identified and deemed to meet the inclusion criteria from review of medical records. A PIS will also be included for the parent of the identified young person. Contact details of the research team are provided in this PIS and families interested in participation are asked to make contact. The study will also be described, and a PIS provided, to young people and their parent(s) attending either paediatric or adult lipid clinics. For families expressing an interest in participation, a meeting or phone call will be arranged with a member of the research team.

### Sample size

This is an exploratory feasibility study and therefore no power calculation has been used to determine sample size because it is not designed or powered to address the effectiveness of the intervention being evaluated [25, 27, 28]. Sample sizes of between 24 and 50 have been recommended for feasibility studies wishing to provide a standard deviation estimation to inform the sample size calculation of a larger RCT [27, 29, 30]. The target sample size of 24 family units in this study has been based upon this recommendation in addition to knowledge of the current local FH population.

### Randomisation and blinding

Randomisation, stratified by study site, will allocate family units to either usual care and waitlist (control) or to the nutrition and physical activity intervention (intervention) on a 1:1 basis. To facilitate recruitment and retention, family units allocated to the control arm will be placed on a waitlist to receive the intervention at the end of the 12-week study period. Randomisation will be carried out by an independent person using prepared, password-protected randomised lists, following obtainment of informed consent to participate. Family units will be notified of their assigned group at research visit two, after baseline data collection. Due to the nature of the intervention, delivery and study design, it is not possible to blind participants, clinical staff or research staff to the randomisation results. Upon completion of the study, participants in the control group will be asked if they had any communication with other young person-parent dyads in the study. This will assess potential intervention contamination bias which will be taken into consideration in the analysis. The anticipated likelihood of this occurring is low due to the infrequency of outpatient appointments (usually once a year) that families attend.

### Intervention

Family units randomised to the intervention group will receive an individualised, family-based nutrition and physical activity intervention developed specifically for this study. It will be delivered to the family at research visit two by a dietitian and will last approximately 1 h. This will be followed up with four phone calls or email correspondence (according to participant preference) at weeks 2, 4, 8, and 11 of the intervention. Age-specific intervention booklets (10–13 years and ≥ 14 years) have been developed for use in the intervention. These will be provided to the participants at research contact two to enable participants to refer to the intervention information during the 12-week intervention and follow-up sessions. If a young person is participating without a parent, the intervention content and delivery will be changed to facilitate this.

#### Intervention content and delivery

The development and content of the intervention has been described in full elsewhere [31], but will be described briefly. Intervention aims to enable participants to achieve the dietary intakes and physical activity levels currently recommended for people with FH. A literature review of the available guidelines for the management of FH was conducted to determine the following targets for daily dietary intakes and weekly physical activity levels:

1. Total fat intake ≤ 30% of total energy intake (TEI)
2. Saturated fat intake of ≤v10% TEI achieved via replacement of saturated fats with monounsaturated and polyunsaturated fats
3. Dietary cholesterol intake ≤ 300 mg
4. Consumption of ≥ 5 portions of fruit and vegetables
5. Age appropriate fibre intake: 10-year-olds = 20 g/day; 11–16 year-olds = 25 g/day and 30 g/day for ≥ 17 years
6. 2 g of plant stanol/sterols
7. Reduce time spent engaged in sedentary behaviours
8. Age appropriate physical activity levels:

- Adults: ≥ 150 min a week of moderate intensity physical activity or ≥ 75 min of vigorous intensity physical activity, or a mixture of the two. Additional activity focussing on improving muscle strength should be undertaken twice a week.
- Young people: ≥ 60 min of moderate-to-vigorous physical activity (MVPA) each day, with three of these sessions each week being of vigorous intensity and including activities that strengthen muscle and bone.

Evidence suggests that behaviour change interventions based on theory are more successful than those developed without a theoretical base [32]. As there are many theoretical models, but no consensus on which one is superior, the theoretical domains framework (TDF) was used in the development of this intervention [33]. The TDF brings together 33 psychological theories that are relevant to behaviour change and sorts them into 84 constructs (the component parts of theories) which have been organised into 14 domains (broader areas in which a theory may be applied, e.g. motivation). This TDF can be used as a framework to identify barriers and facilitators to achieving behaviour change and has been validated for use in developing theoretically informed interventions [33]. To develop an understanding of behavioural factors influencing adherence to treatment in people with FH a qualitative evidence synthesis was conducted by our team. This synthesis identified enablers and barriers to treatment adherence in young people and adults with FH [34] which were then mapped onto the TDF domains. Appropriate behaviour change techniques (BCTs) to target these domains were selected using a 93-item BCT taxonomy [35]. The utility of these BCTs in this intervention were considered in relation to the APPEASE criteria: affordability, practicality, effectiveness, cost-effectiveness, acceptability, side-effects/safety and equity [36]. A total of 26 BCTs have been incorporated into the intervention. The intervention will be delivered to all participants by one of two dietitians.

#### Control group

Family units assigned to the control group will be informed that they are on a waitlist to receive the intervention at the end of the 12-week study period and receive usual care. At the three NHS sites involved, usual care is comprised of an annual appointment with a lipid clinic doctor and continuation on their pharmacological treatment, if applicable. Young people are not offered the opportunity to receive dietetic advice when attending paediatric outpatient visits and young people and adults are given an option to receive advice at their first adult clinic outpatient visit. Therefore, it is unlikely that any participant on the waitlist control will receive dietetic advice during the 12-week intervention period; however, this will be monitored. These family units will not be contacted during the 12-week intervention period, except before week 12 to arrange the endpoint dietary and physical activity assessments ahead of research visit three.

### Primary aim outcomes

#### Feasibility and acceptability outcomes (study objectives 1, 2 and 3)

The feasibility and acceptability will be estimated through recruitment, randomisation and retention rates, attendance at study visits, completion rates of questionnaires, completeness of dietary intake and physical activity data and rates of successful collection of clinical measurements (blood samples, anthropometric measurements and blood pressure).

#### Intervention fidelity and evaluation outcomes (study objective 4)

To explore if the intervention is delivered as intended, the dietitians will complete checklists and record detailed reflections for each intervention and follow-up session. Details of the goals set, and self-reported attainment by participants during follow-up sessions, will be recorded in case report forms (CRFs). Intervention intensity will be estimated through recording the number and duration of any face to face, email and telephone contact with participants. The potential mechanisms of impact will be explored through descriptive analysis of this data, along with qualitative data collection from participants in the intervention group. Exploration of these outcomes will enable the intervention to be refined before utilisation in future studies.

#### Qualitative outcomes (study objective 5)

The acceptability of the intervention and the research methods used will be explored through additional data regarding the acceptability of the intervention and research methodology collected from semi-structured qualitative interviews. The sub-sample selected for interview will comprise of up to 30 participants based on sample sizes in previous similar studies [37–42]. It will also include participants who did not want to take part in the full research study but consented to the qualitative component of the study (Fig. 1). A purposive sampling approach, using a maximum variation method, will be taken to select participants for the sub-sample according to individual characteristics, opinions and experiences relevant to this study [43, 44]. Separate topic guides have been developed for adults and young people to provide some structure to the interviews.

The qualitative data will also be used to help understand how and why the intervention was effective or ineffective and if the intervention will be successful in a wider context [26, 45, 46]. It will also be used to help improve the design and conduct of any future trials [26, 47].

### Secondary aim outcomes

The potential efficacy of the developed intervention upon dietary intakes, physical activity and sedentary time, body composition, CVD risk factors and quality of life will be estimated. This will be done through measurement of changes in a range of clinical and behavioural outcomes before and after the intervention period. Results will also be used to estimate the variability of each outcome measure and to identify the suitability of the outcome tools/measures chosen to detect any changes. When possible, standard deviation of the outcome measure will be calculated to inform the sample size calculation of a future large-scale RCT.

Behavioural outcomes (dietary intake, physical activity, sedentary time) will be assessed in the week immediately preceding research visits two and three (Fig. 1). Anthropometric, clinical and biochemical outcomes, and quality of life measures will be collected at baseline and endpoint assessments conducted at research visits two and three (Fig. 1). Further details of each outcome are provided below and in Table 1.

**Table 1:** Outcome measures, data collection methods and time points

Study aim	Objectives being explored	Outcome measures	Data collection method/tool	Time point collected (weeks)				
				− 1	− 1 to 0	0	11–12	12
Primary: Feasibility and acceptability	1. Will young people and their parent be willing to participate in the proposed family-based intervention? 2. Will families accept randomisation to the control or intervention group, adhere to research methods in their allocated group and complete the study? 3. Are the research methods that are used to capture data fit for purpose?	Recruitment, retention, randomisation and adherence to protocol rates	Screening, recruitment and randomisation logs, monitoring of study visit attendance, successful collection of clinical outcome measures and withdraw/loss to follow up records.	Continuous during data collection period				
	4. Is there sufficient protocol fidelity in the intervention group?	Intervention intensity and adherence to intervention protocol and delivery	Self-completed checklists and recording of the number & duration of contact with participants					
	5. Upon completion of the study, was the overall experience of the intervention and/or the study research processes positive and potentially reproducible in an adequately powered trial?	Self-reported experiences and beliefs of participants regarding the study methodology &/or intervention	Semi-structured qualitative interviews					x
Secondary: Potential efficacy	No specific objectives stated for this secondary study aim	Patient characteristics: medication, education levels, smoking status and FH genetic variant	CRF developed for study	x		x		x
		QoL score (HRQoL and component physical, emotional, social and school functioning scores for young people and single index value and VAS rating for adults)	Age appropriate PedsQL™ QoL inventory or EUROQOL 5D-3L health questionnaire			x		x
		Mean fat, saturated fat, monounsaturated fat, polyunsaturated fat, fibre, plant sterol/stanols, cholesterol and fruit and vegetable intake per day (grams and % of daily energy intake)	INTAKE24 online dietary recall system completed for 4 days		x		x	
		MVPA (min/day)	ActiGraph GT3X+ accelerometer worn for 7 days		x		x	
		Time spent sitting/lying, standing and walking (min/day)	activPAL3 accelerometer worn for 7 days		x		x	
		Self-reported physical activity levels (MET minutes per week and categorised into high, medium or low level groups for adults and assigned score between 1 and 5 for young people, in which 1 indicates low levels and 5 high levels, of PA)	Age appropriate IPAQ, PAQ-A or PAQ-C questionnaire		x		x	
		Anthropometric measurements: height (cm), weight (kg), BMI (kg/m^2^), body composition including body fat (%) and fat free mass (%)	Tanita body composition analyser and stadiometer			x		x
		Systolic and diastolic blood pressure (mmHg)	Mean of two measures by a sphygmomanometer (three measures if 1^st^ and 2^nd^ differ by more than 10mmHg)			x		x
		Serum concentrations (mmol/l) and component lipid breakdown (mmol/l and %) of different classes (very large, large, medium and small) of VLDL, LDL, HDL particles; mean diameter of VLDL, LDL & HDL particles (nm); serum concentration of total cholesterol, LDL, HDL, HDL2, HDL3, remnant cholesterol, triglycerides (mmol/l) and Apolipoproteins (g/l).	Metabolomic analysis of serum samples obtained from processing of 25ml whole blood samples			x		x

*CRF* case report form, *QoL* quality of life, *VAS* visual analogue scale, *MVPA* moderate-to-vigorous physical activity, *IPAQ* International Physical Activity Questionnaire, *PAQ*-*A* the Physical Activity Questionnaire for Adolescents, *PAQ*-*C* the Physical Activity Questionnaire for older Children, *BMI* body mass index, *VLDL* very large density lipoprotein, *LDL* low-density lipoprotein, *HDL* high-density lipoprotein

#### Dietary intake

Participants will record their dietary intake over four non-consecutive days (including one weekend day) using INTAKE24, an online 24-h recall tool. INTAKE24 is a validated, self-completed computerised dietary recall system based on multiple-pass 24-h recall developed specifically for young people. It is a secure tool, accessed by individually issued log-on details. It has been found to produce mean daily energy and nutrient intakes comparable with interviewer-led 24-h recalls in young people aged 11–24 years, at a significantly lower cost [48]. This method offers several benefits over traditional paper-based methods, such as pre-programmed completeness checks, prompts and food photographs to improve data capture. The automated coding system ensures consistency of coding and removes the risk of data entry errors. Furthermore, newer technology-based methods of dietary assessment have been demonstrated to be preferred by young people and adults compared to traditional methods [49–52]. Participants will be asked about their consumption of supplements and plant stanol/sterol fortified foods at research visits 2 and 3.

The data will be analysed to produce mean daily intakes of fat, saturated fat, monosaturated fat, polyunsaturated fat, cholesterol, plant stanols/sterols, fibre and fruits and vegetables.

#### Physical activity levels

Free-living MVPA will be measured using an ActiGraph GT3X+ accelerometer (ActiGraph, Pensacola, USA), worn by participants on an elasticated belt on the waist above the midline of the right thigh for seven consecutive days, except for water-based activities. This is a tri-axial device that detects the frequency and amplitude of acceleration in three axes. Participants will also be asked to complete a physical activity log book, to capture waking/sleeping times and any activities which required the monitor to be taken off. When processing the data, age- and sex-specific cut points will be applied to define sedentary time, light, moderate and vigorous physical activity during waking hours. Total daily time spent in MVPA will be obtained by totalling the duration of all moderate and vigorous physical activity bouts for each day. This value will then be averaged over the number of valid days to determine mean time spent in MVPA per day.

Self-report measures of physical activity have been found to overestimate MVPA in young people [53] and adults [54]. However, self-report and objective measures have not been compared within populations of individuals with FH. Self-report measures are more practical and less burdensome for study participants and may be a more appropriate outcome measure to use in a future larger scale trial. Thus, each participant will also complete age appropriate physical activity self-report questionnaires. For adults, the International Physical Activity Questionnaire (IPAQ) will be used which is a validated self-report tool for measuring physical activity in adults aged 18–64 years old [55]. The Physical Activity Questionnaire for Older Children (PAQ-C) [56] and Adolescents (PAQ-A) [57] will be used with participants aged 10–14 years and 15–18 years old respectively, as recommended by a comprehensive systematic review and expert panel consensus [58]. These questionnaires will be scored to assign individuals into one of five categories which indicate low to high levels of physical activity. These will be compared to the objective MVPA measurements.

#### Sedentary time

This will be measured using an activPAL3 accelerometer (PAL Technologies Ltd., Glasgow, UK) worn on the front of the thigh. The activPAL3 will be made waterproof using a nitrile sleeve and a waterproof hypoallergenic medical dressing will be used to attach it to the leg, enabling participants to wear it continuously for 24 h/day over 7 days. It contains a tri-axial accelerometer which responds to signals related to gravitational forces and provides information on thigh inclination [59]. This allows for precise differentiation between postures in prolonged free-living activities [60] and represents a valid measure of time spent sitting/lying, standing and walking in adults [61, 62] and young people [63–65]. Total daily time spent sitting/lying, standing and walking will be obtained for each day and will be averaged over the number of valid days across all waking hours.

#### Participant characteristics

Information about smoking status, education, medication and FH genetic variation will be obtained from participants and used to characterise the intervention and control groups.

#### Anthropometric and other clinical measurements

Height and weight will be measured to determine body mass index (BMI). Resting systolic and diastolic blood pressure will be estimated by averaging two separate measurements using a sphygmomanometer. Blood pressure and BMI have been chosen as outcome measures due to research finding these to be independently associated with CVD risk in adults with FH [66]. There has been no similar research carried out in young people with FH; however, BMI and blood pressure have been reported to independently influence future CVD risk in young adults without FH [67].

Body composition will be measured using bioelectrical impedance (Tanita™ MC-780MA) with attention paid specifically to fat and fat-free mass. While no research to date has investigated the role of these outcomes measures upon CVD risk in individuals with FH, they have been found to influence the serum blood lipid levels of young people without FH [68]. Therefore, these measures will help to characterise the participants in the control and intervention groups and allow for interpretation of any changes in serum lipid levels observed in participants before and after the intervention period. Furthermore, changes in these outcomes have been found to moderate the influence of physical activity interventions upon blood lipid measures in adults without FH [69, 70] highlighting the need for future research to control for such measures.

#### Quality of life

The need for further research to explore the potential detrimental impacts that individuals with FH may experience as a result of making changes to their diet and physical activity levels was highlighted in a Cochrane systematic review [20]. Therefore, the Quality of life (QoL) of all participants will be measured.

Young person participants will be asked to complete an age-appropriate Pediatric Quality of Life Inventory ™ (PedsQL™) Version 4.0. The PedsQL 4.0 Generic Core Scales will be used which have been validated for measuring the health-related quality of life (HRQoL) of young people aged 2 to 18 years with chronic health conditions [71]. This multidimensional self-report scale consists of 23 items, covering four domains of HRQoL: physical, emotional, social and school. The completed inventories will be scored using the recommended scoring system [72] to produce a score for HRQoL and a breakdown of the component physical, emotional, social and school functioning scores.

Adult participants will be asked to complete an EuroQol Group EQ-5D-3L health questionnaire as recommended by NICE [73] and validated for use in European adults [74]. Participants will be asked to rate their degree of impairment in five health domains (mobility, self-care, usual activities, pain/discomfort and anxiety/depression) using one of three responses (no problems, some problems and extreme problems). The recommended scoring system will be used [75] which produces a five-digit health state profile representative of five dimensions of health. This will then be converted into a single index value using a validated value set created for use in the UK [76]. Participants will also be asked to rate their perceived health by drawing a line to a point on a visual analogue scale (VAS) between the endpoints of ‘best imaginable health state’ and ‘worst imaginable health state’. The VAS data will be presented as a single value between 1 and 100.

#### Blood lipidomic markers

The primary aim in the treatment of FH is a reduction in LDL-C concentrations [15]. However, it is recognised that focussing on a single lipid marker does not adequately reflect an individual’s overall risk of CVD or the clinical effect of any pharmacological or lifestyle treatment [17, 77]. Therefore, several lipidomic markers have been selected as outcome measures (Table 1), although these may change as the literature develops. These have been chosen on the basis of their proposed role in the prediction and development of CVD risk [17, 78] and/or sensitivity to change via dietary intakes and physical activity as proposed in previous research [77, 79].

A maximum of 25 ml whole blood will be collected from each participant which is within the safe limits advised by the World Health Organisation (WHO) for use in child health research [80]. Blood samples will be collected, stored and analysed in accordance with the Human Tissue Act 2004. Participants will be requested to fast for a minimum of 6 h and to avoid strenuous physical activity for 12 h prior to sample collection.

Samples will be sent to the laboratory of the hospital in which they were collected and will be processed according to the laboratory Standard Operating Procedures and under the supervision of the laboratory team. Once centrifuged, the sample will be split whereby some of the sample will be analysed immediately to determine the full lipid profile (LDL-C, HDL and total cholesterol) in the hospital laboratory. The remainder of the sample will be stored at − 80 °C as plasma in anonymously labelled microtubes in the hospital laboratory prior to transport, contained in dry ice, to the University of Bristol MRC Integrative Epidemiology Unit (IEU) Metabolomics Facility. These samples will undergo batch metabolomic analysis at the end of the data collection period to determine the levels of lipidomic markers.

### Data collection

#### Quantitative data

All quantitative participant data will be collected on anonymised purpose-designed CRFs or questionnaires. These data will then be inputted into a study database (Microsoft Access) by a member of the research team. The database is password-protected and accessible only by the chief and co-investigators.

Data from the activPAL and ActiGraph GT3X+ accelerometers will be downloaded using custom software (PAL Technologies Ltd., Glasgow, UK and ActiGraph, Pensacola, USA, respectively). Raw data from the ActiGraph GT3X+ accelerometer will be downloaded and analysed using the manufacturer’s software (ActiLife software v6.13.4; ActiGraph, Pansacola, FL, USA). The activPAL3 data will be downloaded using the manufacturer’s software (PAL software v7.2.38; PAL Technologies Ltd., Glasgow, UK), and the raw data will be cleaned and processed using a validated algorithm [81]. The participant’s physical activity log-books will be used to aid identification of non-wear periods and sleep time; these periods will be removed from subsequent analysis. Data collected using the INTAKE24 online tool will be downloaded and analysed manually using Excel and statistical software.

#### Qualitative data

The audio-recorded qualitative data collected will be transcribed verbatim by a member of the research team, or an individual from a University of Bristol approved transcription company, and stored using NVivo (NVivo10, QSR International, 2012) data management software.

### Withdrawal

Each participant will have the right to withdraw from the study at any time. The study design aims to minimise attrition by not overburdening participants but if there is significant attrition (loss of more than 15 individuals) over the study period, additional individuals with similar characteristics to those lost will be recruited. Participants will be withdrawn from the study if their hospital FH care team feel that their participation is negatively affecting their clinical status. This includes participants who become pregnant during the study as they will be required by necessity to stop statin treatment. A member of the hospital FH care team will inform the research dietitian of these cases. In all cases, data collected up until the point of withdrawal will be included in the analyses, unless the participant expresses a wish for their data to be destroyed. If a reason for withdrawal is given, this will be documented in the participants CRF.

### Analysis plan

Data collected from young people will be analysed separately from the data collected for adult parent participants.

#### Quantitative data

Recruitment, randomisation and retention rates, attendance at study visits, completion rates of questionnaires, completeness of dietary intake and physical activity data, rates of successful collection of clinical measurements and categorisation results of self-reported physical activity levels will be descriptively analysed. The other quantitative variables of interest (Table 1) are continuous. Therefore, where possible, groups will be summarised by means and standard deviations (S.D.) for (i) baseline, (ii) end point assessment and (iii) for the changes over the intervention period. This will be carried out for individuals in the control and intervention groups.

Comparisons will be made between the endpoint assessment means in the intervention and control groups with or without adjustment for the baseline values and stratification, as appropriate. 95% confidence intervals (C.I.) will be reported for mean differences. Comparisons may be made between the intervention and control groups in respect of the mean changes from baseline if this is found to improve precision [82].

Additionally, the dietary intake and physical activity data at both baseline and endpoint assessment will be compared to the target levels determined for the intervention (outlined previously in ‘intervention content’) and will be descriptively analysed for participants in the control and intervention groups.

#### Qualitative data

Interview transcripts will be analysed using thematic analysis [83, 84]. NVivo (NVivo10, QSR International, 2012) data management software will be used to manage the transcript data. Thematic analysis involves coding the interview transcripts, using an inductive approach. This step will be carried out by two members of the research team who will independently code data on a sub-sample of the data set. The analysts will then discuss their initial coding and endeavour to reach consensus about the codes assigned with the aim of developing a definitive coding frame for the complete data set. Once all transcripts have been coded inductively, the research team members will work deductively to group codes into themes associated with the research objectives of this study. A descriptive, narrative approach will then be taken to explore the findings in the context of the trial research aims and their experiences of participation in the trial. The findings will be presented using anonymised participant quotes to illustrate any identified themes.

### Harms

There are no known risks to the participants in this study. Any adverse event, regardless of relationship with the study treatment, will be recorded and reported in accordance with University Hospitals Bristol NHS Foundation Trust and the University of Bristol (as sponsor) guidance.

### Data monitoring

A Data and Safety Monitoring Board has not been formally established, due to the low risk nature of the intervention. All adverse events will be reported to the chief investigator (FJK) and reviewed at monthly meetings with co-investigators (JPHS and FEL) to determine whether the study should be discontinued because of participant safety.

### Auditing

The University of Bristol, as Sponsor of this study, has a Service Level Agreement in place with University Hospitals Bristol NHS Foundation Trust. As part of this agreement, University Hospitals Bristol NHS Foundation Trust will undertake monitoring of research projects where University of Bristol is fulfilling the responsibilities of Research Sponsor. A minimum of 10% of projects will be monitored. If this study is selected for audit, it would be carried out independently to the study team and Sponsor.

### Ethics and Dissemination

#### Research ethics approval

The study has been approved by the South West-Cornwall & Plymouth Research Ethics Committee (REC) (Reference: 18/SW/0121).

#### Protocol amendments

Any amendments to the protocol will be submitted to the REC and Health Research Authority (HRA) for approval, after permission is sought from the Sponsor. Only once the amendment has been approved by the REC and HRA and has received confirmation of continued capacity and capability from NHS sites (or acknowledged in the case of a minor amendment) will the amended protocol be implemented. Deviations to the protocol outlined in the published protocol will be highlighted in any subsequent publication of the study findings.

#### Consent and assent

Written informed consent is required from all participants to participate in this study. For young persons aged 10–15 years, this will be obtained from their parent or guardian, with written informed assent also collected from the young person. Informed consent will be sought when patients attend the hospital for a routine clinic appointment or at the first research visit, prior to commencing any data collection. A member of the research team will be responsible for the consent process. There will be an opportunity to discuss any remaining questions the participant may have with a member of the research team prior to signing the consent or assent form. A copy of the signed assent and/or consent form will be filed in the participants’ medical records to ensure that the hospital FH care teams are aware of their involvement in the study. Eligible individuals who decline participation in the study will be offered the option to undertake the qualitative component of the research study only. These individuals will receive a different PIS outlining what is involved in this part of the study. These individuals will be required to give informed consent or assent prior to collection of any data.

All individuals will be advised that participation in the study is voluntary and that they have the right to withdraw at any time, without the need for explanation and that their decision will not impact upon the care they receive.

#### Confidentiality

All data will be anonymised, with participants identified by a unique study number. Confidentiality will be ensured through the application of the University of Bristol information security and data handling policies. These are compliant with the Data Protection Act 2018 and conform to the security standards of the NHS IG toolkit. All participant information (i.e. names, addresses, dates of birth etc.) will be stored separately from data collected during the study (i.e. dietary intake) and participants will be identified by a unique study number. Personal identifiable paper records will be stored separately from anonymised paper records and CRFs. All paper records will be stored in secure storage facilities in University Hospitals Bristol, Royal United Bath Hospital or St. George’s Hospitals NHS trust sites. No personal data will leave the sites until archiving at the end of the study. Electronic data will be stored securely using password-protected databases on a University of Bristol computer in the NIHR Biomedical Research Centre (Nutrition Theme), Level 3 University Hospitals Bristol NHS Trust Education and Research Centre. This is a secure site within the Trust. The University of Bristol computer hard drive is also encrypted. Only members of the study team will have access to the dataset.

#### Dissemination policy

Summaries of preliminary findings will be produced for study participants and the individual results for each participant will be recorded in their medical records.

Manuscripts reporting the findings will be produced for publication in relevant, peer-reviewed international journals. As this is a feasibility study, the results will be used to inform and develop a larger RCT, if appropriate, which will be adequately powered to detect clinically significant changes in outcome measures.

## Patient and public involvement

Two events have taken place with patient and public involvement:

1. Seven individuals, three young people and four adults, with FH under the care of the University Hospitals Bristol NHS foundation Trust have participated in the development work underpinning this research. They gave feedback about the initial design of the intervention and data collection methods.
2. Ten young people (aged 11–17 years) from the Bristol Young Persons Advisory Group (YPAG) also participated in the development work underpinning this research. This group is comprised of young people who are interested in healthcare and research who meet regularly in Bristol to help researchers with their projects. This group reviewed and gave feedback on the participant facing documents, data collection methods and details of the intervention involved with this study.

## Discussion

Dietary intakes and physical activity are considered key components of managing the CVD risk for individuals with FH. However, there is a recognised lack of research to support the efficacy of adhering to the current guidelines for promoting a favourable CVD risk factor profile. Furthermore, the best approach to promote behavioural changes in this population is yet to be determined. This paper describes the protocol of a pilot, randomised waitlist-controlled trial designed to estimate the feasibility and acceptability of conducting a family-based nutrition and physical activity intervention to young people, and their affected parent, with FH. The data obtained in this trial could be used to inform the development of a future clinical trial powered to detect clinically significant changes in the chosen outcome measures, if deemed appropriate.

### Study status

The study is now closed for recruitment and data collection was completed in March 2020.

## Supplementary information

**Additional file 1.** SPIRIT 2013 statement checklist: recommended items to address in a clinical trial protocol and related documents.

## Data Availability

Not applicable.

## Abbreviations

FH: Familial hypercholesterolaemia
LDL-C: Low-density lipoprotein cholesterol
CVD: Cardiovascular disease
HCP: Healthcare professional
RCT: Randomised controlled trial
MRC: Medical Research Council
SPIRIT: Standard protocol items: recommendations for interventional trials
NHS: National Health Service
PIS: Participant information sheet
TEI: Total energy intake
MVPA: Moderate-to-vigorous physical activity
TDF: Theoretical domains framework
BCT: Behaviour change technique
CRF: Case report form
IPAQ: International physical activity questionnaire
PAQ-C: The physical activity questionnaire for older children
PAQ-A: The physical activity questionnaire for adolscents
BMI: Body mass index
QoL: Quality of life
HRQoL: Health-related quality of life
NICE: National Institute for Health and Care Excellence
VAS: Visual analogue scale
WHO: World Health Organisation
IEU: Integrative Epidemiology Unit
CI: Confidence intervals
REC: Research Ethics Committee
HRA: Health Research Authority
YPAG: Young Persons Advisory Group
